# Biogenic amine reduction by food additives in *Cheonggukjang*, a Korean fermented soybean paste, fermented with tyramine-producing heterogeneous bacterial species

**DOI:** 10.1016/j.heliyon.2024.e26135

**Published:** 2024-02-09

**Authors:** Dabin Kim, Young Hun Jin, Jae-Hyung Mah

**Affiliations:** Department of Food and Biotechnology, Korea University, Sejong 30019, Republic of Korea

**Keywords:** Biogenic amines, Fermented soybean foods, Fermentation, Glycine, Sodium benzoate, Potassium sorbate

## Abstract

This study was conducted to mitigate the food safety risks related to biogenic amine (BA) by reducing the BA content in *Cheonggukjang* using applicable food additives. In *in*-*vitro* experiments, of the additives tested, tartaric acid (TA), potassium sorbate (PS), and sodium benzoate (SB) considerably inhibited tyramine production of strains of *Bacillus* spp. and *Enterococcus faecium* while less affecting their growth. In addition to these three additives, two additives, glycine (GL) and nicotinic acid (NA), reported to have significant inhibitory effects in previous studies, were applied to the *Cheonggukjang* fermentation with prolific tyramine-producing strains of *B. subtilis* and *E. faecium*. The content of tyramine in the *Cheonggukjang* samples treated with TA, PS, SB, GL, and NA was significantly reduced by 27.5%, 50.7%, 51.4%, 76.1%, and 100.0%, respectively, compared to the control sample. Additionally, the content of polyamines (putrescine, cadaverine, spermidine, and spermine) in the GL-treated sample was reduced by 42.6%–62.4%. The mode of action could be attributed to inhibiting the bacterial decarboxylase activity and/or growth. Consequently, excluding NA that interfered with *Cheonggukjang* fermentation, GL was the most outstanding additive with an inhibitory effect on tyramine formation in food, followed by SB and PS, all of which showed a more than 50% reduction. Therefore, the use of appropriate additives could be one of the promising strategies to avoid the food safety issues implicated in BAs in *Cheonggukjang*.

## Highlights

• All applicable food additives were tested to inhibit biogenic amine (BA) production.
• Three organic acids inhibited tyramine production *in vitro*, but not bacterial growth.
• In *Cheonggukjang*, glycine, sodium benzoate, and potassium sorbate were most effective.
• Glycine was the most effective in reducing tyramine formation in *Cheonggukjang*.
• Additives could mitigate the food safety risks associated with BAs in *Cheonggukjang*.

## 1 Introduction

Biogenic amines (BAs) are commonly produced via enzymatic decarboxylation of amino acids and the reductive amination and transamination of aldehydes and ketones [1]. BAs play important roles in biological systems and cell processes, such as modulating neuronal activity and synaptic transmission, synthesizing nucleic acids and proteins, and stabilizing cellular membranes [2,3]. Despite the biological functions of BAs, deficiency or inhibition of amine oxidases metabolizing BAs as well as excessive intake of BAs may result in various adverse health effects, including hypo- and hypertension, abdominal pain, nausea, vomiting, sweating, and hot flushes [2,4]. Histamine and tyramine are particularly the most harmful BAs causing foodborne diseases such as scombroid poisoning (i.e., histamine poisoning) and cheese crisis (i.e., tyramine toxicity), respectively [5]. Histamine and tyramine can also induce a synergistic effect resulting in greater cytotoxicity of tyramine [6]. Furthermore, putrefactive polyamines are recognized to increase the toxicity of the two harmful BAs by interfering with amine oxidases’ detoxification capabilities [7]. Considering these adverse health effects implicated in BAs, several governments and international organizations have recommended guidance levels for histamine in some fish species known to be high in histamine content and processed foods made from these fish species [4]. Although governments or international organizations have not established regulations on BAs other than histamine, various studies have described the cytotoxicity threshold of BAs as follows: tryptamine, 80.10 mg/kg; putrescine, 881.5 mg/kg; cadaverine, 510.9 mg/kg; histamine 440.6 mg/kg; tyramine 301.8 mg/kg; spermidine, 1452.5 mg/kg; spermine, 653.6 mg/kg in foods [[8], [9], [10], [11], [12]].

*Cheonggukjang*, one of the most famous Korean fermented soybean pastes, is known to date back to the ancient kingdom of Korea, Goguryeo (also referred to as Koguryo) (BC 37–AD 668) [13]. At present, *Cheonggukjang* is produced by either traditional or modern methods using rice straw rich in *Bacillus* spp. or starter cultures, respectively [[14], [15], [16]]. During the fermentation process, *Bacillus* spp. degrades proteins and carbohydrates in soybeans, the *Cheonggukjang* raw material, with their enzymatic activity, giving the food a unique savory taste, aroma, and sticky mucilage [17,18]. Since sticky mucilage contains poly-γ-glutamic acid and isoflavone compounds, *Cheonggukjang* can inhibit atherosclerosis, accelerate aging and inflammation, and enhance anticancer activity and immune function [[19], [20], [21], [22]]. Despite the nutritional benefits, several studies have reported the potential risks implicated in the BA content in *Cheonggukjang*. According to a previous study on BA content in retail *Cheonggukjang*, tyramine and β-phenylethylamine were detected at levels of 457.42 ± 573.15 and 36.22 ± 29.55 mg/kg, respectively [23]. Such levels exceeded the toxicity limits for tyramine (100 mg/kg) and β-phenylethylamine (30 mg/kg) suggested by Ten Brink et al. [12]. In another study on the content of BA in various regional *Cheonggukjang* in South Korea, the two vasoactive BAs were detected at 103.85 ± 78.46 and 99.29 ± 129.23 mg/kg, respectively [15], exceeding the toxicity limits. *Cheonggukjang* is made from soybeans rich in protein, which are degraded into amino acids used as precursors for BA formation; it contains numerous prolific BA-producing microorganisms such as *B. subtilis* and *Enterococcus faecium*, and probably other species belonging to these genera as well [4,15,23]. As such, the high BA content in *Cheonggukjang* seems to be an inevitable risk. In previous studies, strategies applied to reduce such a high BA content in *Cheonggukjang* mainly include the use of starter cultures [[24], [25], [26]]. However, studies comparing the controlling effects of all applicable food additives on the formation of BAs by diverse bacterial species in *Cheonggukjang* are lacking.

Therefore, the current study was conducted to search for food additives that inhibit BA formation effectively and are applicable to *Cheonggukjang*. For this, food additives were selected from those listed in both the Codex Alimentarius regional standard for fermented soybean paste in Asia [27] and the Food Additives Code of South Korea [28], and tested for their inhibitory effects on the toxic vasoactive BA (i.e., tyramine) production of *B. subtilis* and *E. faecium* strains reported to be prolific BA producers. The practical impacts of selected food additives on BA content changes as well as the microbiological and physicochemical characteristics of *Cheonggukjang* during fermentation, were also investigated. This study may provide guidance for the food industry that aims to produce *Cheonggukjang* with reduced BA content.

## 2 Materials and methods

### 2.1 Microbial strains and preparation of their suspension for *Cheonggukjang* fermentation

The *Bacillus* type strains, *B. licheniformis* KCTC 1918 and *B. subtilis* KCTC 3135, obtained from the Korea Collection for Type Cultures (KCTC; Daejeon, South Korea) and the BA-degrading *B. licheniformis* CH7P22 originated from *Cheonggukjang* [25] served as negative controls since they have incapacity to produce BAs. *B. subtilis* CB 9-4 strain capable of producing tyramine [15] was used for the determination of controlling effects of additives on tyramine production in this study. All the strains of *Bacillus* were cultured in tryptic soy broth (TSB; Difco, Becton Dickinson, Sparks, MD, USA) at 37 °C for 24 h and stored as a glycerol stock (final concentration of 20%, *v*/*v*) at −70 °C until further use.

The *Enterococcus* type strain, *E. faecium* KCCM 12118, obtained from the Korean Culture Center of Microorganisms (KCCM, Seoul, South Korea) and an isolated prolific strain of tyramine producer, *E. faecium* CH5H28, isolated from *Cheonggukjang* in our preliminary tests were also used for the determination of the controlling effects of additives on tyramine production. The two *Enterococcus* strains were cultured in de Man, Rogosa, and Sharpe (MRS, Laboratorios Conda, Madrid, Spain) broth at 37 °C for 48 h and stored under the same conditions as previously described.

The microbial suspension of the strains of each genus was prepared following the protocol described by Park et al. [23]. The two strains, *B. subtilis* CB 9-4 and *E. faecium* CH5H28, were used for *Cheonggukjang* fermentation as they produced higher amounts of tyramine than other strains of each genus used in this study. The microbial counts of each suspension were adjusted to 8 Log CFU/mL for *B. subtilis* CB 9-4 and 6 Log CFU/mL for *E. faecium* CH5H28, and the suspensions were stored at 4 °C and used within 2 days.

### 2.2 Determination of the controlling effects of food additives on BA production

Applicable additives were selected based on the Codex Alimentarius regional standard for fermented soybean paste in Asia [27] and the Food Additives Code provided by the Ministry of Food and Drug Safety in South Korea [28]. Subsequently, five food additives, including l-tartaric acid (TA), potassium sorbate (PS) as a substitute for sorbic acid, sodium benzoate (SB) as a substitute for benzoic acid, sodium polyphosphate (SP), and lactic acid (LA) were selected. Additionally, glycine (GL) and nicotinic acid (NA), which had demonstrated significant controlling effects on BA formation in previous research [[29], [30], [31]], were also tested for fermentation experiments. All food additives were obtained from Serim Food (Bucheon, South Korea).

In order to examine the controlling effects of food additives on BA production, the assay media were prepared as following procedures. In TSB (for *Bacillus* spp.) or MRS broth (for *E. faecium* strains), 0.5% of l-tyrosine disodium salt hydrate, l-histidine monohydrochloride monohydrate, l-ornithine monohydrochloride, and l-lysine monohydrochloride as well as 0.0005% pyridoxal-HCl (all from Sigma-Aldrich Chemical Co., St. Louis, MO, USA) were added. Then, each food additive was added to each broth and the final concentrations were adjusted to 0.5, 1.0, and 5.0% for TA, SP, and LA, or 0.06, 0.1, 0.5, 1.0, and 5.0% for PS and SB. The pH of the broths was adjusted to 5.80 by adding 2 mol/L hydrochloric acid (Sigma). Thereafter, the broths were autoclaved at 121 °C for 15 min for TSB and 121 °C for 12 min for MRS broth, which are hereafter referred to as “assay media”.

One hundred microliters of the glycerol stock of each *Bacillus* spp. or *E. faecium* strains (see Section 2.1) were transferred to 5 mL of TSB or MRS broth, respectively, and incubated aerobically at 37 °C for 24 h (for *Bacillus* spp.) or 48 h (for *E. faecium* strains). Then, 100 μL of the cultures was inoculated in respective fresh broths. Following the second incubation under the same conditions, 200 μL of the cultures were transferred to the assay media (the final bacterial concentrations were about 6 log CFU/mL) and incubated under the same conditions. The assay medium inoculated with the culture, but with no additive served as the control. Cultured assay media after incubation were sampled for the measurements of the microbial counts (see Section 2.4) as well as the BA content (see Section 2.5).

### 2.3 Fermentation of *Cheonggukjang* treated with the selected food additives

In order to investigate the practical impacts of the selected food additives on the reduction of tyramine and other BAs, *Cheonggukjang* fermentation was conducted following the procedure described by Park et al. [23]. Considering that BA-producing heterologous bacteria such as *E. faecium* and *B. subtilis* are commonly present in retail *Cheonggukjang* products, bacterial inocula (prepared in Section 2.1) were inoculated in 200 g of steam-sterilized soybeans. The final microbial counts in the inoculated samples were about 6 log CFU/g for *B. subtilis* CB 9-4 and 4 log CFU/g for *E. faecium* CH5H28.

Seven types of the samples were made as follows: B sample (blank, noninoculated sample prepared without food additives), C sample (control, inoculated sample without food additives), TA sample (inoculated sample containing 0.5% TA), PS sample (inoculated sample containing 0.1% PS), SB sample (inoculated sample containing 0.06% SB), GL sample (inoculated sample containing 1.0% GL), and NA sample (inoculated sample containing 1.0% (inoculated sample with 0.1% NA). Fermentation of *Cheonggukjang* were carried out at 37 °C for 4 days. Twenty grams of soybeans in each *Cheonggukjang* sample were sampled daily to determine the food quality parameters (see Section 2.4) and BA content (see Section 2.5).

### 2.4 Measurements of food quality parameters

Similar to the previous study, the physicochemical properties of the samples, including pH and water activity (a_w_), were measured [23]. The pH of the samples was measured with Orion 3-star pH Benchtop (Thermo Scientific, Waltham, MA, USA), while the a_w_ were determined using AquaLab Pre (Meter Group, Inc., Pullman, WA, USA).

The microbiological properties of samples were also measured following the procedures conducted by Park et al. [23]. Plate count agar (PCA, Difco) and m-Enterococcus Agar (m-EA; MB Cell, Seoul, South Korea) were used to determine total viable mesophilic bacterial counts and enterococcal counts, respectively. The concentrations of each bacterium in the samples were measured by counting colony-forming units (CFU) grown on plates containing approximately 10–300 colonies from the respective media [32] and adjusting for dilution.

### 2.5 Measurement of BAs in assay media and *Cheonggukjang* samples

The measurement of BA content in assay media and *Cheonggukjang* samples (prepared in Sections 2, 2.2.3) was carried out following the procedures described by Park et al. [23]. The procedures were as follows. (i) preparation of standard BAs and extraction of BAs from assay media and *Cheonggukjang* samples, (ii) derivatization of BAs, (iii) chromatographic separation of BAs. The limit of detection (LOD) and limit of quantitation (LOQ) for all BAs were determined by Yoon et al. [33], and both levels were as follows. LOD and LOQ for standard solutions and assay media: approximately 0.10 μg/mL, LOD and LOQ for food matrices: less than 0.10 mg/kg and 0.31 mg/kg, respectively.

### 2.6 Statistical analysis

*In vitro* BA production tests were performed in duplicate, while fermentation experiments and all physicochemical and microbiological measurements were performed in triplicate. Data were presented as the mean ± standard deviation of duplicates or triplicates. Significant differences were determined using one-way analysis of variance (ANOVA) with Fisher's pairwise comparison using Minitab statistical software (Version 17.1.0. Minitab Inc., State College, PA, USA). Differences with a probability (*p*) value of <0.05 were considered statistically significant.

## 3 Results and discussion

### 3.1 Controlling effects of food additives on tyramine production of *Enterococcus* and *Bacillus* strains in assay media

Food additives tested to inhibit toxic vasoactive BA (i.e., tyramine) production by prolific tyramine-producing *Bacillus* and *Enterococcus* strains which had been reported in previous studies [15,25] were selected based on no or less effect on *Cheonggukjang* flavor among those listed in both the Codex Alimentarius regional standard for fermented soybean paste in Asia [27] and the Food Additives Code of South Korea [28]. The selected food additives were TA, SP, LA, PS, and SB, and their inhibitory effects were determined through *in-vitro* experiments.

As shown in Table 1, Table 2, LA exhibited a moderate growth inhibitory effect (*p* < 0.05) on *B. subtilis* and *E. faecium* strains at concentrations of at least 1.0% and 5.0%, respectively. However, *B. licheniformis* CH7P22, which was deemed suitable as a BA-degrading starter and/or protective culture in a previous study [25], was unable to grow at even the lowest concentration (0.5%) of LA tested. *Bacillus* spp. and *E. faecium* strains generally produced less tyramine in response to increasing LA concentration. At the highest concentration (5.0%), tyramine production by the majority of *Bacillus* strains and all *E. faecium* strains was completely inhibited. At the lowest LA concentration tested (0.5%), *E. faecium* CH5H28 showed a higher tyramine production than the control (*p* < 0.05).

**Table 1:** Effects of food additives on tyramine production of *Bacillus* spp. and *E. faecium* strains in assay media.

Treatments	Bacterial tyramine production (μg/mL)				
	*B. subtilis* KCTC 3135	*B. subtilis* CB9-4	*B. licheniformis* CH7P22	*E. faecium* KCCM 12118	*E. faecium* CH5H28
Control a	0.15 ± 0.21 b^,A^	1.94 ± 0.88 ^AB^	0.19 ± 0.01 ^AB^	145.22 ± 8.25 ^A^	127.14 ± 1.75 ^A^
Tartaric acid					
TA 0.5%	0.16 ± 0.22 ^A^	1.71 ± 0.20 ^AB^	0.21 ± 0.07 ^A^	122.24 ± 2.34 ^BCD^	119.71 ± 3.64 ^AB^
TA 1.0%	0.29 ± 0.08 ^A^	1.39 ± 0.02 ^AB^	0.22 ± 0.08 ^A^	107.68 ± 5.00 ^DEF^	118.60 ± 3.95 ^AB^
TA 5.0%	ND c^,A^	1.70 ± 0.00 ^AB^	0.19 ± 0.07 ^AB^	104.51 ± 2.74 ^EF^	118.09 ± 5.71 ^AB^
Sodium polyphosphate					
SP 0.5%	0.14 ± 0.20 ^A^	1.76 ± 0.25 ^AB^	0.20 ± 0.02 ^AB^	109.72 ± 4.97 ^CDE^	117.37 ± 2.53 ^AB^
SP 1.0%	ND ^A^	1.68 ± 0.05 ^AB^	0.22 ± 0.04 ^A^	95.84 ± 3.10 ^FG^	116.28 ± 7.22 ^ABC^
SP 5.0%	ND ^A^	1.39 ± 0.00 ^AB^	0.21 ± 0.13 ^AB^	101.95 ± 2.68 ^EF^	105.01 ± 4.90 ^BCD^
Lactic acid					
LA 0.5%	0.40 ± 0.56 ^A^	1.60 ± 0.39 ^AB^	ND ^D^	93.43 ± 8.75 ^FG^	132.50 ± 7.88 ^A^
LA 1.0%	0.03 ± 0.04 ^A^	1.64 ± 0.00 ^AB^	ND ^D^	81.43 ± 8.05 ^G^	99.96 ± 1.13 ^CD^
LA 5.0%	ND ^A^	1.30 ± 0.00 ^AB^	ND ^D^	ND ^I^	ND ^F^
Potassium sorbate					
PS 0.06%	0.09 ± 0.13 ^A^	2.06 ± 0.04 ^A^	0.18 ± 0.04 ^AB^	132.11 ± 4.62 ^B^	104.10 ± 0.08 ^BCD^
PS 0.1%	0.01 ± 0.01 ^A^	1.77 ± 0.00 ^AB^	0.18 ± 0.03 ^AB^	115.04 ± 0.13 ^CDE^	94.65 ± 3.57 ^D^
PS 0.5%	ND ^A^	1.71 ± 0.40 ^AB^	0.17 ± 0.01 ^ABC^	37.62 ± 1.76 ^H^	20.72 ± 0.54 ^E^
PS 1.0%	ND ^A^	1.25 ± 0.00 ^AB^	0.15 ± 0.01 ^ABC^	ND ^I^	ND ^F^
PS 5.0%	ND ^A^	1.16 ± 0.00 ^AB^	0.07 ± 0.10 ^CD^	ND ^I^	ND ^F^
Sodium benzoate					
SB 0.06%	ND ^A^	1.69 ± 0.14 ^AB^	0.14 ± 0.03 ^ABC^	117.77 ± 1.76 ^BCD^	96.48 ± 8.07 ^D^
SB 0.1%	ND ^A^	1.64 ± 0.08 ^AB^	0.10 ± 0.06 ^BCD^	124.96 ± 1.35 ^BC^	96.24 ± 8.99 ^D^
SB 0.5%	ND ^A^	1.52 ± 0.00 ^AB^	ND ^D^	52.79 ± 4.46 ^H^	3.07 ± 0.10 ^F^
SB 1.0%	ND ^A^	1.27 ± 0.00 ^AB^	ND ^D^	ND ^I^	ND ^F^
SB 5.0%	ND ^A^	1.00 ± 0.00 ^B^	ND ^D^	ND ^I^	ND ^F^

a The assay medium without food additives served as control.

b Data represent mean ± standard deviation determined by duplicate experiments. Mean values with different upper letters (A-I) in the same columns are significantly different (*p* < 0.05).

c ND: Not detected.

**Table 2:** Effects of food additives on bacterial growth of *Bacillus* spp. and *E. faecium* strains in assay media.

Treatments	Total mesophilic viable bacterial counts (log CFU/mL)			Enterococcal counts (log CFU/mL)	
	*B. subtilis* KCTC 3135	*B. subtilis* CB9-4	*B. licheniformis* CH7P22	*E. faecium* KCCM 12118	*E. faecium* CH5H28
Control a	7.66 ± 0.03 b^,A^	7.70 ± 0.11 ^AB^	8.00 ± 0.02 ^A^	9.06 ± 0.04 ^A^	8.62 ± 0.01 ^A^
Tartaric acid					
TA 0.5%	7.63 ± 0.13 ^A^	7.63 ± 0.02 ^AB^	7.28 ± 0.61 ^AB^	8.89 ± 0.05 ^AB^	8.70 ± 0.07 ^A^
TA 1.0%	7.38 ± 0.05 ^AB^	7.66 ± 0.15 ^AB^	6.80 ± 0.77 ^BC^	8.91 ± 0.09 ^AB^	8.63 ± 0.07 ^A^
TA 5.0%	5.95 ± 0.00 ^D^	4.75 ± 0.21 ^C^	4.74 ± 0.03 ^D^	8.98 ± 0.06 ^AB^	8.73 ± 0.07 ^A^
Sodium polyphosphate					
SP 0.5%	7.85 ± 0.01 ^A^	8.13 ± 0.31 ^A^	7.36 ± 0.05 ^AB^	9.05 ± 0.03 ^A^	8.80 ± 0.04 ^A^
SP 1.0%	7.61 ± 0.03 ^A^	7.42 ± 0.01 ^AB^	7.41 ± 0.05 ^AB^	8.99 ± 0.07 ^A^	8.72 ± 0.00 ^A^
SP 5.0%	5.00 ± 0.00 ^EF^	3.98 ± 0.16 ^D^	6.01 ± 0.09 ^C^	8.58 ± 0.06 ^CD^	8.71 ± 0.04 ^A^
Lactic acid					
LA 0.5%	7.67 ± 0.08 ^A^	7.17 ± 0.08 ^B^	ND c^,E^	8.71 ± 0.07 ^BC^	8.33 ± 0.25 ^B^
LA 1.0%	2.52 ± 0.01 ^H^	5.14 ± 0.13 ^C^	ND ^E^	8.15 ± 0.21 ^E^	7.72 ± 0.17 ^C^
LA 5.0%	2.46 ± 0.06 ^H^	5.34 ± 0.08 ^C^	ND ^E^	6.00 ± 0.00 ^G^	5.76 ± 0.03 ^D^
Potassium sorbate					
PS 0.06%	6.76 ± 0.40 ^BC^	7.02 ± 0.03 ^B^	7.67 ± 0.06 ^AB^	8.80 ± 0.02 ^ABC^	8.52 ± 0.04 ^AB^
PS 0.1%	6.24 ± 0.02 ^CD^	4.72 ± 0.34 ^C^	7.08 ± 0.08 ^B^	8.35 ± 0.04 ^DE^	8.38 ± 0.03 ^AB^
PS 0.5%	4.85 ± 0.02 ^F^	4.87 ± 0.04 ^C^	4.19 ± 0.03 ^D^	7.13 ± 0.07 ^F^	5.50 ± 0.07 ^D^
PS 1.0%	4.03 ± 0.06 ^G^	5.02 ± 0.09 ^C^	ND ^E^	6.07 ± 0.03 ^G^	5.54 ± 0.04 ^D^
PS 5.0%	3.98 ± 0.09 ^G^	5.08 ± 0.43 ^C^	ND ^E^	5.63 ± 0.10 ^H^	5.56 ± 0.08 ^D^
Sodium benzoate					
SB 0.06%	6.72 ± 0.34 ^BC^	7.24 ± 0.09 ^B^	7.12 ± 0.06 ^AB^	8.88 ± 0.00 ^AB^	8.13 ± 0.02 ^BC^
SB 0.1%	5.63 ± 0.46 ^DE^	4.98 ± 0.03 ^C^	6.16 ± 0.08 ^C^	8.85 ± 0.04 ^ABC^	8.36 ± 0.11 ^AB^
SB 0.5%	4.85 ± 0.00 ^F^	4.87 ± 0.04 ^C^	ND ^E^	5.52 ± 0.05 ^H^	5.35 ± 0.49 ^D^
SB 1.0%	3.83 ± 0.09 ^G^	4.80 ± 0.28 ^C^	ND ^E^	ND ^I^	5.31 ± 0.19 ^D^
SB 5.0%	3.96 ± 0.09 ^G^	4.80 ± 0.14 ^C^	ND ^E^	ND ^I^	ND ^E^

a The assay medium without food additives served as control.

b Data represent mean ± standard deviation determined by duplicate experiments. Mean values with different upper letters (A-I) in the same columns are significantly different (*p* < 0.05).

c ND: Not detected.

Similarly, PS and SB also showed inhibitory effects on the growth of *Bacillus* spp. and *E. faecium* strains in a concentration-dependent manner, completely suppressing that of *B. licheniformis* CH7P22, especially at concentrations greater than 1.0%. Furthermore, SB completely hindered the growth of all *E. faecium* strains at the highest concentration (5.0%). Both PS and SB generally showed significant inhibitory effects (*p* < 0.05) on tyramine production of *Bacillus* spp. and *E. faecium* strains probably (but almost certainly) due to such high antimicrobial effects. Notably, PS exhibited complete inhibition of tyramine production of all *E. faecium* strains, although it inhibited bacterial growth weaker than SB (*p* < 0.05), which completely inhibited growth at the highest concentration (5.0%).

*Bacillus* spp. exhibited a concentration-dependent decrease in growth in the presence of TA and SP, whereas the growth of *E. faecium* strains remained constant or decreased marginally. TA and SP significantly inhibited growth (*p* < 0.05) at the highest concentration tested (5.0 %). *Bacillus* spp. and *E. faecium* strains both tended to decrease their tyramine production as TA and SP concentrations increased. Interestingly, TA and SP appeared to inhibit tyramine production of *E. faecium* strains in a manner similar to PS, i.e., these three additives significantly inhibited tyramine production while slightly inhibiting bacterial growth. Some food additives (TA and NA) have been reported to suppress tyrosine decarboxylase activity (rather than bacterial growth) to inhibit *E. faecium* tyramine production [34,35]. The three additives, TA, SP, and PS, are, therefore, likely to have similar inhibition modes to the reported additives. Conversely, inhibition of tyramine production by the other additives LA and SB may be mainly due to their antimicrobial activity, which is a characteristic of organic acids (or their salts).

In conclusion, the food additives inhibited the tyramine production of *E. faecium* strains (except SP) and exhibited concentration-dependent antimicrobial activity against *Bacillus* spp. and *E. faecium* strains. Therefore, it would be of interest for future research to investigate the various mechanisms by which food additives inhibit tyramine production and/or possess antimicrobial activity, as well as the susceptibility of various bacterial species to additives.

### 3.2 Effects of food additives on parameters of food quality and content of tyramine and other BAs in *Cheonggukjang* with *Bacillus* and *Enterococcus* strains

To evaluate the practical controlling effect of food additives on tyramine formation, *in situ* experiments were performed during *Cheonggukjang* fermentation. As BA-producing heterogeneous bacteria such as *E. faecium* and *B. subtilis* are commonly present in *Cheonggukjang* products [4,15,23], prolific tyramine-producing strains *E. faecium* CH5H28 and *B. subtilis* CB 9-4 were co-inoculated into intact *Cheonggukjang* samples. Food additives to inhibit BA formation in *Cheonggukjang* during fermentation were selected according to two arbitrary criteria: (1) They should not lower bacterial cell counts by more than 1.5 Log CFU/mL from the initial count, and (2) They should concentration-dependently inhibit tyramine production of *E. faecium* strains. Consequently, three food additives (TA, PS, and SB) were selected. The PS and SB concentrations were set at the maximum levels in food in which each additive is used, as specified by the MFDS (0.1% for PS and 0.06% for SB), while the TA concentration was selected to be 0.5%, a concentration tested *in vitro* in Section 3.1, which falls within the acidity range (0.25–0.61%) of commercial *Cheonggukjang* products [26]. Additionally, GL and NA, which had demonstrated significant controlling effects on BA formation of *Bacillus* [31] and *Enterococcus* strains [29], respectively, in previous research, were also tested at the lowest concentrations at which inhibitory effects had been reported in fermentation experiments (1.0% for GL and 0.1% for NA) as well as *in-vitro* tests. Therefore, in this study, these additives were applied to *Chenggukjang* fermentation experiments without *in vitro* testing.

#### 3.2.1 Effects of food additives on food quality parameters

**Fig. 1:**
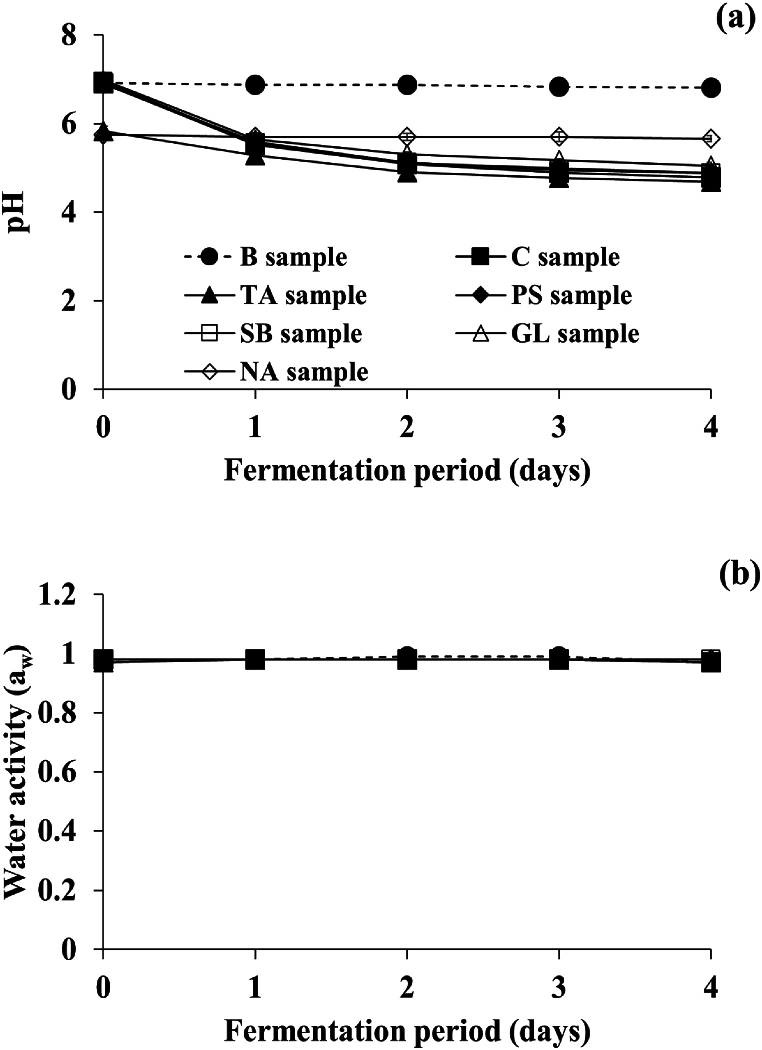
Effects of food additives on physicochemical properties during fermentation of *Cheonggukjang* inoculated with prolific tyramine-producing *B. subtilis* CB 9-4 and *E. faecium* CH5H28. (a) pH, (b) water activity. ●: B sample prepared without inocula nor food additives (blank), ■: C sample prepared with inocula, but without food additives (control), ▲: TA sample prepared with both inocula and 0.5% tartaric acid, ◆: PS sample prepared with both inocula and 0.1% potassium sorbate, □: SB sample prepared with both inocula and 0.06% sodium benzoate, △: GL sample prepared with both inocula and 1.0% glycine, ◇: NA sample prepared with both inocula and 0.1% nicotinic acid. Error bars indicate standard deviations determined from triplicate experiments.

Food quality parameters were measured to examine if *Cheonggukjang* samples treated with each of the food additives were fermented properly. The initial pH values of most samples (B, C, PS, SB, and GL samples) were determined to be 6.93 ± 0.03 (mean ± standard deviation of the values of those samples), as shown in Fig. 1. Subsequently, the pH values thereof (except for the B sample) decreased steadily to 4.86 ± 0.13 at the end of the fermentation, which may be due to the production of lactic acid by *E. faecium* [23]. Somewhat differently, the initial pH values of the TA and NA samples were determined to be 5.83 and 5.75, respectively, lower than those of the other samples (*p* < 0.05). This observation may be attributed to the acidic properties of TA and NA. Similar to other samples, the pH value of the TA sample decreased to 4.69 ± 0.02 until fermentation ends. However, that of the NA sample stayed constant over the fermentation due to slower increases in the total viable mesophilic bacterial counts and enterococcal counts compared to other samples.

**Fig. 2:**
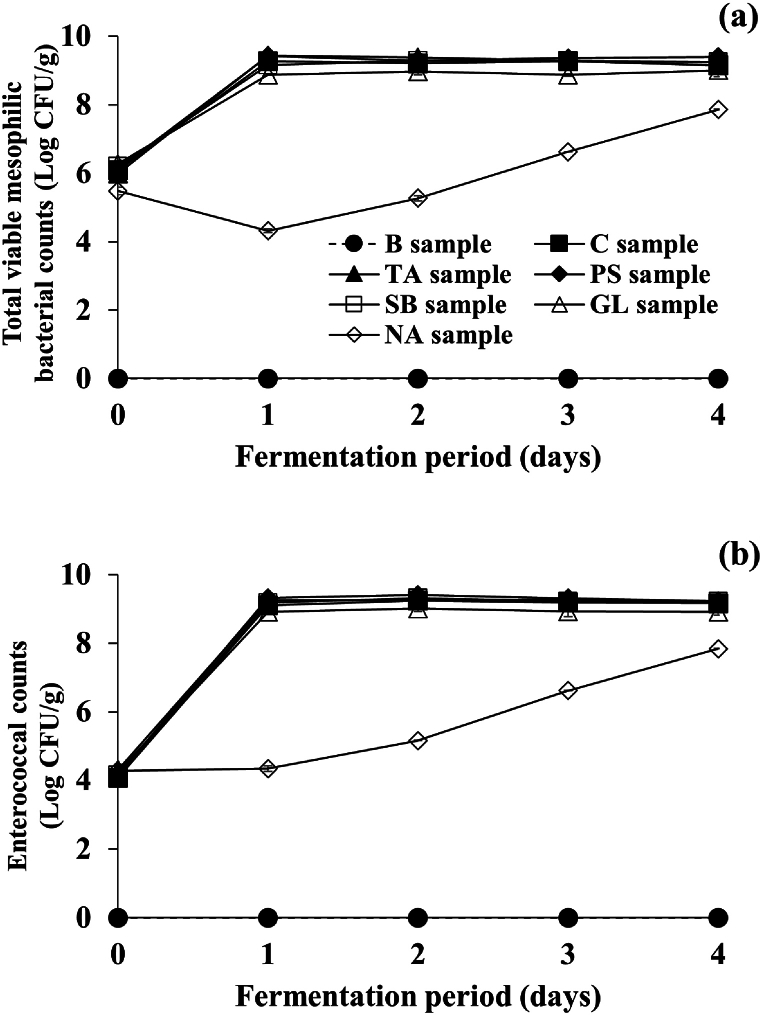
Effects of food additives on microbiological properties during fermentation of *Cheonggukjang* inoculated with prolific tyramine-producing *B. subtilis* CB 9-4 and *E. faecium* CH5H28. (a) Total viable mesophilic bacterial counts, (b) enterococcal counts. ●: B sample prepared without inocula nor food additives (blank), ■: C sample prepared with inocula, but without food additives (control), ▲: TA sample prepared with both inocula and 0.5% tartaric acid, ◆: PS sample prepared with both inocula and 0.1% potassium sorbate, □: SB sample prepared with both inocula and 0.06% sodium benzoate, △: GL sample prepared with both inocula and 1.0% glycine, ◇: NA sample prepared with both inocula and 0.1% nicotinic acid. Error bars indicate standard deviations determined from triplicate experiments.

Total viable mesophilic bacterial and enterococcal counts in all inoculated samples began at about 6 and 4 log CFU/g, respectively (Fig. 2). By day 1, the both counts in the majority of samples (with the exception of the NA sample) increased to about 9 log CFU/g, respectively, and stayed constant until fermentation ends. However, the total mesophilic bacterial counts in the NA sample decreased considerably to 4.32 ± 0.05 log CFU/g by day 1 and gradually increased to 7.86 ± 0.01 log CFU/g until fermentation ends. In comparison, the enterococcal counts gradually increased to 7.84 ± 0.01 log CFU/g at the end of the fermentation without a decrease in the middle of fermentation. Such results could be interpreted by speculation that NA inhibits the growth of *Bacillus* more than *Enterococcus*. To support this speculation, Koser and Kasai [35] reported that adding NA of more than 0.1% in a synthetic medium significantly inhibited the growth of *B. subtilis* and completely restricted that of *B. megaterium*. However, another study showed that the treatment with 0.1% NA slightly inhibited *Enterococcus* growth [29]. In the current study, no *Bacillus* colonies were detected on PCA plates for measuring total viable mesophilic bacterial counts in the NA sample on the first day (data not shown). Furthermore, on the last day of the fermentation, the number of *Bacillus* colonies was lower than the detection limit (<10 colonies = 2 log CFU/g) suggested by the International Organization for Standardization [29]. Thus, the total viable mesophilic bacterial counts detected from the first day to the end of the fermentation can be ascribed almost exclusively to *E. faecium*. This study demonstrates that NA inhibits the growth of *B. subtilis* in *Cheonggukjang*, in contrast to Kang et al. [29], who found that *B. subtilis* thrived in NA-treated *Cheonggukjang*. The susceptibility of *B. subtilis* strains to NA must be investigated further, as such disparate results are likely due to differences in the *B. subtilis* strains used.

Furthermore, in all samples, water activity was determined to be approximately 0.97–0.98 throughout the fermentation (Fig. 1). Considering the overall changes in food quality parameters of the samples, it appears that fermentation of most samples treated with the tested food additives, except for the NA sample, proceeded suitably.

#### 3.2.2 Controlling effects of food additives on BA formation

**Fig. 3:**
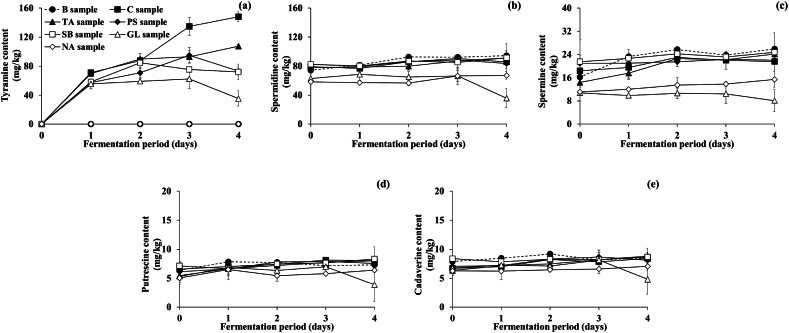
Effects of food additives on BA content during fermentation of *Cheonggukjang* inoculated with prolific tyramine-producing *B. subtilis* CB 9-4 and *E. faecium* CH5H28. (a) Tyramine, (b) spermidine, (c) spermine, (d) putrescine, (e) cadaverine. Other BAs, including tryptamine, β-phenylethylamine, and histamine, were not detected. ●: B sample prepared without inocula nor food additives (blank), ■: C sample prepared with inocula, but without food additives (control), ▲: TA sample prepared with both inocula and 0.5% tartaric acid, ◆: PS sample prepared with both inocula and 0.1% potassium sorbate, □: SB sample prepared with both inocula and 0.06% sodium benzoate, △: GL sample prepared with both an inocula and 1.0% glycine, ◇: NA sample prepared with both inocula and 0.1% nicotinic acid. Error bars indicate standard deviations determined from triplicate experiments.

After evaluating the food quality parameters, it was determined if the BA concentrations in *Cheonggukjang* treated with each additive were significantly reduced. Fig. 3 depicts variations in the concentrations of not only tyramine (the most abundant and toxicologically significant BA) but also putrescine, cadaverine, spermidine, and spermine. The content of other BAs, such as tryptamine, β-phenylethylamine, and histamine, were also measured (as shown in Supplementary Table 1), but they will not be discussed further because they were not detected during fermentation.

As shown in Fig. 3a, tyramine was not detected in all samples before fermentation (day 0). The content in the B sample showed no change over fermentation period (certainly due to the absence of inoculation of tyramine-producing strains), while that in the C sample increased significantly and steadily to 148.17 ± 7.01 mg/kg until fermentation ends, exceeding the toxicity limit for tyramine (100 mg/kg) suggested by Ten Brink et al. [12]. The high level of tyramine in the C sample was likely associated with the tyramine production of *B. subtilis* and *E. faecium* strains, which is in consistent with the observations from previous studies [15,23,25]. It is well-known that naturally fermented *Cheonggukjang* products contain these tyramine-producing bacteria [4,15,23]. Based on previous and current studies, tyramine accumulation in *Cheonggukjang* (particularly naturally fermented varieties) appears inevitable. Therefore, the tyramine content in *Cheonggukjang* is a critical topic that must be reduced in some way, including the strategy proposed in this study.

Throughout fermentation, tyramine was not detected in the NA sample. As described in Section 3.2.1, although NA significantly inhibited the growth of both *B. subtilis* and *E. faecium,* the enterococcal counts in the NA sample were still high enough to produce tyramine. These results indicate that NA suppresses the activity of enzymes (particularly tyrosine decarboxylase) involved in formation of tyramines. In order to support this speculation, Kang et al. [29] reported the inhibitory effect of NA on tyrosine decarboxylase activity derived from *E. faecium*. In addition to the inhibition of tyramine formation, the NA sample was not properly fermented due to almost complete inhibition of *B. subtilis,* which plays an important role in *Cheonggukjang* fermentation. Notably, Kang et al. [29] reported that a *B. subtilis* strain grew well in *Cheonggukjang* treated with NA, which is somewhat different from the current study. As discussed in Section 3.2.1, such distinguished susceptibility of *Bacillus* strains to NA may be strain-dependent. Therefore, susceptibility testing of *Bacillus* strains against NA is required to apply NA to *Cheonggukjang* fermentation.

Similar to the C sample, the tyramine content in the TA sample gradually increased to 107.50 ± 2.51 mg/kg until fermentation ends. However, the content was significantly lower than in the C sample (*p* < 0.05). After the second day of fermentation, compared to the C sample, tyramine content in the TA sample showed a reduction by 27.5% until fermentation ends. Although the tyramine concentration in *Cheonggukjang* still exceeded the tyramine toxicity limit (100 mg/kg), the addition of TA could be used as part of hurdle technology to inhibit tyramine formation. There have been no reports of TA inhibiting the formation of tyramine in foods. Previous studies have reported BA reduction using other organic acids such as acetic, citric, and lactic acid [34,36] due to their acidification and/or antimicrobial activity. However, bacterial growth in the TA sample was comparable to that in the C sample, indicating that TA restricted tyramine formation in *Cheonggukjang* by inhibiting tyrosine decarboxylase activity rather than the bacterial growth. To support this supposition, Kang et al. [29] reported that TA inhibited *E. faecium*-derived tyrosine decarboxylase activity slightly.

In terms of inhibition of tyramine formation in *Cheonggukjang*, GL showed the strongest effect, followed by SB and PS. In all the GL, SB, and PS samples, the tyramine content increased considerably until the middle of the fermentation (by day 1, 2, or 3 depending on each additive; for the GL sample, a minor increase by day 3) and significantly decreased thereafter (*p* < 0.05). In detail, the content of the GL sample gradually increased to 62.46 ± 13.46 mg/kg on day 3 and decreased to 35.45 ± 10.80 mg/kg until fermentation ends. In the SB sample, the tyramine content steadily increased to 84.68 ± 7.45 mg/kg on day 2 and decreased to 72.04 ± 10.79 mg/kg until fermentation ends. Similar to the GL sample, the content of tyramine in the PS sample gradually increased to 94.95 ± 10.68 mg/kg on day 3 and decreased to 73.12 ± 5.88 mg/kg until fermentation ends. As described previously, the additives differently revealed the onset, duration, and intensity of the inhibitory effect on tyramine formation. Consequently, GL was the most efficient additive for reducing tyramine during fermentation. PS and SB inhibited tyramine formation significantly on the second and third days, respectively, although they were less effective than GL (*p* < 0.05). Considering that the *Cheonggukjang* fermentation period can vary based on consumer preference [37], the tyramine formation in *Cheonggukjang* can be effectively suppressed by applying each of the three additives selectively based on the preferred fermentation period.

At the end of fermentation, the tyramine concentrations in the GL, SB, and PS samples were reduced by 76.1, 51.4, and 50.7%, respectively, compared to the C sample (*p* < 0.05). As stated previously, GL exhibited the greatest inhibitory effect compared to SB and PS. However, it is important to note that the additive concentrations used in this study varied for the reasons outlined in Section 3.2. Notably, the results of this study do not describe the absolute effects of the additives at the same concentration, which may not be applicable depending on the additives. As described in Section 3.1, the suppressive effects of SB and PS on tyramine production in the assay media were predicted to be due to antimicrobial activity and inhibition of tyrosine decarboxylase activity, respectively. In fermentation experiments, as described in Section 3.2.1, however, the bacterial growth in the GL, SB, and PS samples (treated with GL, SB, and PS, respectively) was comparable to that in the C and TA samples, indicating that the effects of these three additives were attributed to inhibition of tyrosine decarboxylase activity rather than antimicrobial activity. Consistent with the results of fermentation experiments, previous research suggests that GL and PS (as its acid form, sorbic acid) inhibit tyrosine decarboxylase activity *in vitro* to reduce tyramine content [33,34]. SB's inhibitory mechanism has not been reported in the scientific literature, but in this study, it was anticipated that the inhibition mechanisms would differ between *in-vitro* (assay media) and fermentation experiments. Consequently, there is a need for additional research into the disparities between the primary inhibition mechanisms resulting from differences in menstrua.

Regarding putrescine, cadaverine, spermidine, and spermine (Fig. 3 b, c, d, and e), the NA sample was analyzed but was not described hereafter because it was not adequately fermented as described in Section 3.2.1. In most samples (B, C, TA, PS, and SB samples), the initial content of putrescine, cadaverine, and spermidine was determined to be 5.46 ± 1.20 to 7.15 ± 0.75, 6.71 ± 0.99 to 8.40 ± 0.42, and 74.32 ± 5.05 to 82.79 ± 3.59 mg/kg, respectively, and remained constant thereafter. In contrast, the initial content of the BAs in the GL sample was 5.34 ± 1.38, 6.75 ± 0.90, and 63.00 ± 6.31 mg/kg, respectively, remained constant on day 3, and significantly decreased to 3.88 ± 2.88, 4.83 ± 2.59, and 35.91 ± 13.44 mg/kg, respectively, until fermentation ends. Meanwhile, the initial spermine content in most samples (the B, C, TA, PS, and SB samples) was determined to be 14.36 ± 1.57 to 21.51 ± 7.58 mg/kg and stayed constant or somewhat increased until fermentation ends. However, in the GL sample, the initial content was significantly lower (10.80 ± 2.80 mg/kg) than in the other samples (*p* < 0.05) and slightly decreased to 8.13 ± 3.87 mg/kg until fermentation ends. Notably, the use of GL in the *Cheonggukjang* fermentation effectively inhibited the formation of polyamines such as putrescine, cadaverine, spermidine, and spermine by 51.7, 42.6, 58.2, and 62.4%, respectively, compared to those in the C sample (*p* < 0.05). Such an inhibitory effect could result from the suppression of amino acid decarboxylase activity, as suggested by Jin et al. [30] and Mah and Hwang [31]. Additionally, the GL and NA sample contained significantly lower levels of spermidine and spermine, suggesting that GL and NA play a role in metabolizing the polyamines into other compounds. However, the exact mechanism(s) of the reduction of BA by GL and NA is still unclear.

Altogether, the tested additives, besides NA, which was excluded due to improper fermentation, exhibited inhibitory effects on the formation not only of tyramine but also of other BAs (putrescine, cadaverine, spermidine, and spermine) and would be useful additives to avoid food safety issues implicated in BAs (especially tyramine) in *Cheonggukjang*.

## 4 Conclusions

The presence of indigenous microorganisms capable of producing BAs inevitably leads to BA accumulation in food (particularly naturally fermented varieties). Many previous studies have reported the BA formation of autochthonous bacteria such as *B. subtilis* and *E. faecium* present in naturally fermented *Cheonggukjang*. In this study on *Cheonggukjang*, out of all applicable food additives, TA, PS, and SB greatly inhibited tyramine production *in vitro*, less affecting the bacterial growth. These three additives, along with two additives (GL and NA) reported in previous studies, were applied in *Cheonggukjang* fermentation experiments. As a result, TA, PS, SB, GL, and NA showed considerable tyramine reduction rates of 27.5, 50.7, 51.4, 76.1, and 100.0%, respectively, compared to the C sample. Furthermore, other polyamines (putrescine, cadaverine, spermidine, and spermine) were reduced by 51.7, 42.6, 58.2, and 62.4% in the GL sample, respectively. Therefore, PS, SB, and GL were found to be highly effective in reducing the BA content in *Cheonggukjang* by more than 50% reduction, whereas NA, which inhibits fermentation, was not considered an effective additive.

The selected food additives have been approved for use in accordance with the Codex Alimentarius regional standard for fermented soybean paste in Asia [27] and the Food Additives Code of South Korea [28]. However, in reality, the use of food additives has been avoided because they may affect the organoleptic properties of *Cheonggukjang*. Despite this current situation, the current study implies that food additives need to be used to mitigate food safety risks implicated in BAs, while considering the sensory characteristics of the food. Therefore, this study may provide guidance for the food industry that aims to produce *Cheonggukjang* (and other fermented soybean foods) with reduced BA content.

## Funding

This work was supported by the National Research Foundation of Korea (NRF) grant funded by the Korea Government (MSIT) (no. 2020R1I1A3052118).

## Data availability

The datasets generated during and/or analyzed during the current study are available from the corresponding author on reasonable request.

## CRediT authorship contribution statement

**Dabin Kim:** Writing – original draft, Investigation, Formal analysis. **Young Hun Jin:** Writing – review & editing, Investigation. **Jae-Hyung Mah:** Writing – review & editing, Supervision, Funding acquisition, Conceptualization.

## Declaration of competing interest

The authors declare that they have no known competing financial interests or personal relationships that could have appeared to influence the work reported in this paper.
