# Supplementary material for: Biogenic amine reduction by food additives in Cheonggukjang, a Korean fermented soybean paste, fermented with tyramine-producing heterogeneous bacterial species
Source: Heliyon. 2024 Feb 9;10(4):e26135. doi: 10.1016/j.heliyon.2024.e26135 (PMC10877360; doi:10.1016/j.heliyon.2024.e26135)
Supplement: Multimedia component 1 [file mmc1.docx]

**Table S1.** Statistical comparison of BA content changed during fermentation of *Cheonggukjang* inoculated with prolific tyramine producing *B. subtilis* CB 9-4 and *E. faecium* CH5H28 under treatments with food additives.

| BA content (mg/kg) | Day ^2^ | Experimental samples ^1^ | | | | | | | |
| --- | --- | --- | --- | --- | --- | --- | --- | --- | --- |
|  |  | B sample | C sample | TA sample | PS sample | SB sample | GL sample | NA sample |  |
| Tryptamine |  |  |  |  |  |  |  |  |  |
|  | 0 | ND ^3,A,a^ | ND ^A,a^ | ND ^A,a^ | ND ^A,a^ | ND ^A,a^ | ND ^A,a^ | ND ^A,a^ |  |
|  | 1 | ND ^A,a^ | ND ^A,a^ | ND ^A,a^ | ND ^A,a^ | ND ^A,a^ | ND ^A,a^ | ND ^A,a^ |  |
|  | 2 | ND ^A,a^ | ND ^A,a^ | ND ^A,a^ | ND ^A,a^ | ND ^A,a^ | ND ^A,a^ | ND ^A,a^ |  |
|  | 3 | ND ^A,a^ | ND ^A,a^ | ND ^A,a^ | ND ^A,a^ | ND ^A,a^ | ND ^A,a^ | ND ^A,a^ |  |
|  | 4 | ND ^A,a^ | ND ^A,a^ | ND ^A,a^ | ND ^A,a^ | ND ^A,a^ | ND ^A,a^ | ND ^A,a^ |  |
| β-phenylethylamine |  |  |  |  |  |  |  |  |  |
|  | 0 | ND ^A,a^ | ND ^A,a^ | ND ^A,a^ | ND ^A,a^ | ND ^A,a^ | ND ^A,a^ | ND ^A,a^ |  |
|  | 1 | ND ^A,a^ | ND ^A,a^ | ND ^A,a^ | ND ^A,a^ | ND ^A,a^ | ND ^A,a^ | ND ^A,a^ |  |
|  | 2 | ND ^A,a^ | ND ^A,a^ | ND ^A,a^ | ND ^A,a^ | ND ^A,a^ | ND ^A,a^ | ND ^A,a^ |  |
|  | 3 | ND ^A,a^ | ND ^A,a^ | ND ^A,a^ | ND ^A,a^ | ND ^A,a^ | ND ^A,a^ | ND ^A,a^ |  |
|  | 4 | ND ^A,a^ | ND ^A,a^ | ND ^A,a^ | ND ^A,a^ | ND ^A,a^ | ND ^A,a^ | ND ^A,a^ |  |
| Putrescine |  |  |  |  |  |  |  |  |  |
|  | 0 | 6.40 ± 0.31 ^4,C,a^ | 6.57 ± 1.05 ^B,a^ | 5.46 ± 1.20 ^B,a^ | 6.15 ± 1.58 ^A,a^ | 7.15 ± 0.75 ^A,a^ | 5.34 ± 1.38 ^A,a^ | 5.06 ± 0.29 ^A,a^ |  |
|  | 1 | 7.85 ± 0.01 ^A,a^ | 7.05 ± 0.07 ^AB,ab^ | 6.65 ± 0.01 ^AB,ab^ | 6.60 ± 0.88 ^A,ab^ | 6.66 ± 0.24 ^A,ab^ | 6.87 ± 1.45 ^A,ab^ | 6.51 ± 1.74 ^A,b^ |  |
|  | 2 | 7.74 ± 0.13 ^A,a^ | 7.41 ± 0.36 ^AB,ab^ | 7.21 ± 0.51 ^A,ab^ | 7.81 ± 0.10 ^A,a^ | 7.49 ± 0.09 ^A,ab^ | 6.36 ± 0.84 ^A,bc^ | 5.46 ± 0.97 ^A,c^ |  |
|  | 3 | 7.18 ± 0.12 ^B,ab^ | 8.14 ± 0.39 ^A,a^ | 7.74 ± 0.06 ^A,a^ | 7.93 ± 0.08 ^A,a^ | 7.66 ± 0.78 ^A,a^ | 7.00 ± 1.38 ^A,ab^ | 5.80 ±0.27 ^A,b^ |  |
|  | 4 | 7.31 ± 0.05 ^B,a^ | 8.03 ± 0.49 ^AB,a^ | 8.03 ± 0.11 ^A,a^ | 7.57 ± 0.72 ^A,a^ | 8.33 ± 2.10 ^A,a^ | 3.88 ± 2.88 ^A,b^ | 6.41 ± 0.30 ^A,ab^ |  |
| Cadaverine |  |  |  |  |  |  |  |  |  |
|  | 0 | 7.90 ± 0.47 ^B,ab^ | 6.71 ± 0.99 ^B,bc^ | 6.36 ± 0.45 ^C,bc^ | 7.07 ± 0.08 ^C,abc^ | 8.40 ± 0.42 ^A,a^ | 6.75 ± 0.90 ^A,bc^ | 6.29 ± 0.78 ^A,c^ |  |
|  | 1 | 8.47 ± 0.04 ^AB,a^ | 7.02 ± 0.29 ^AB,ab^ | 7.17 ± 0.79 ^BC,ab^ | 7.20 ± 0.06 ^BC,ab^ | 7.85 ± 0.81 ^A,ab^ | 7.29 ± 0.47 ^A,ab^ | 6.24 ± 1.46 ^A,b^ |  |
|  | 2 | 9.17 ± 0.49 ^A,a^ | 8.25 ± 0.02 ^AB,b^ | 7.50 ± 0.25 ^BC,bc^ | 8.32 ± 0.32 ^AB,ab^ | 8.29 ± 0.00 ^A,b^ | 7.12 ± 0.66 ^A,cd^ | 6.47 ± 0.29 ^A,d^ |  |
|  | 3 | 8.18 ± 0.38 ^AB,ab^ | 7.85 ± 0.13 ^AB,ab^ | 8.23 ± 0.10 ^AB,ab^ | 8.68 ± 0.06 ^A,a^ | 8.22 ± 1.65 ^A,ab^ | 8.16 ± 1.25 ^A,ab^ | 6.60 ± 0.75 ^A,b^ |  |
|  | 4 | 8.54 ± 0.44 ^AB,a^ | 8.42 ± 0.92 ^A,a^ | 8.84 ± 0.62 ^A,a^ | 8.12 ± 0.97 ^ABC,a^ | 8.67 ± 1.50 ^A,a^ | 4.83 ± 2.59 ^A,b^ | 7.07 ± 0.70 ^A,ab^ |  |
| Histamine |  |  |  |  |  |  |  |  |  |
|  | 0 | ND ^A,a^ | ND ^A,a^ | ND ^A,a^ | ND ^A,a^ | ND ^A,a^ | ND ^A,a^ | ND ^A,a^ |  |
|  | 1 | ND ^A,a^ | ND ^A,a^ | ND ^A,a^ | ND ^A,a^ | ND ^A,a^ | ND ^A,a^ | ND ^A,a^ |  |
|  | 2 | ND ^A,a^ | ND ^A,a^ | ND ^A,a^ | ND ^A,a^ | ND ^A,a^ | ND ^A,a^ | ND ^A,a^ |  |
|  | 3 | ND ^A,a^ | ND ^A,a^ | ND ^A,a^ | ND ^A,a^ | ND ^A,a^ | ND ^A,a^ | ND ^A,a^ |  |
|  | 4 | ND ^A,a^ | ND ^A,a^ | ND ^A,a^ | ND ^A,a^ | ND ^A,a^ | ND ^A,a^ | ND ^A,a^ |  |
| Tyramine |  |  |  |  |  |  |  |  |  |
|  | 0 | ND ^A,a^ | ND ^D,a^ | ND ^D,a^ | ND ^C,a^ | ND ^C,a^ | ND ^C,a^ | ND ^A,a^ |  |
|  | 1 | ND ^A,c^ | 70.96 ± 1.91 ^C,a^ | 69.83 ± 4.03 ^C,a^ | 57.37 ± 4.37 ^B,b^ | 58.33 ± 3.83 ^B,b^ | 55.64 ± 6.74 ^AB,b^ | ND ^A,c^ |  |
|  | 2 | ND ^A,d^ | 87.72 ± 0.88 ^B,a^ | 89.91 ± 7.16 ^B,a^ | 70.91 ± 6.84 ^B,b^ | 84.68 ± 7.45 ^A,a^ | 59.21 ± 3.70 ^A,c^ | ND ^A,d^ |  |
|  | 3 | ND ^A,d^ | 134.97 ± 12.37 ^A,a^ | 92.88 ± 8.31 ^B,b^ | 94.95 ± 10.68 ^A,b^ | 75.38 ± 3.18 ^A,bc^ | 62.46 ± 13.46 ^A,c^ | ND ^A,d^ |  |
|  | 4 | ND ^A,e^ | 148.17 ± 7.01 ^A,a^ | 107.50 ± 2.51 ^A,b^ | 73.12 ± 5.88 ^B,c^ | 72.04 ± 10.79 ^AB,c^ | 35.45 ± 10.80 ^B,d^ | ND ^A,e^ |  |
| Spermidine |  |  |  |  |  |  |  |  |  |
|  | 0 | 74.32 ± 5.05 ^C,ab^ | 79.12 ± 5.99 ^B,a^ | 77.96 ± 7.31 ^B,a^ | 82.79 ± 3.59 ^A,a^ | 82.50 ± 1.86 ^A,a^ | 63.00 ± 6.31 ^A,bc^ | 58.33 ± 2.20 ^A,c^ |  |
|  | 1 | 81.52 ± 2.31 ^BC,a^ | 77.00 ± 3.31 ^B,ab^ | 78.74 ± 0.40 ^B,a^ | 79.52 ± 2.11 ^A,ab^ | 80.28 ± 2.61 ^A,a^ | 68.71 ± 8.07 ^A,b^ | 57.24 ± 0.10 ^A,c^ |  |
|  | 2 | 92.67 ± 3.90 ^A,a^ | 85.98 ± 3.15 ^AB,ab^ | 80.07 ± 5.39 ^AB,b^ | 86.26 ± 2.68 ^A,ab^ | 87.04 ± 2.83 ^A,ab^ | 64.98 ± 8.11 ^A,c^ | 56.71 ± 3.40 ^A,c^ |  |
|  | 3 | 92.27 ± 0.93 ^AB,a^ | 90.49 ± 2.64 ^A,a^ | 86.93 ± 4.34 ^AB,a^ | 88.69 ± 6.57 ^A,a^ | 85.30 ± 8.91 ^A,a^ | 66.52 ± 11.81 ^A,b^ | 66.51 ± 7.29 ^A,b^ |  |
|  | 4 | 94.43 ± 6.83 ^A,a^ | 85.91 ± 4.08 ^AB,ab^ | 91.55 ± 0.76 ^A,a^ | 83.64 ± 7.23 ^A,ab^ | 91.55 ± 19.00 ^A,a^ | 35.91 ± 13.44 ^B,c^ | 67.15 ± 5.09 ^A,b^ |  |
| Spermine |  |  |  |  |  |  |  |  |  |
|  | 0 | 16.24 ± 1.49 ^B,ab^ | 18.42 ± 0.84 ^A,ab^ | 14.36 ± 1.57 ^C,ab^ | 20.94 ± 2.86 ^A,a^ | 21.51 ± 7.58 ^A,a^ | 10.80 ± 2.80 ^A,b^ | 11.13 ± 0.26 ^B,b^ |  |
|  | 1 | 23.31 ± 2.46 ^A,a^ | 19.54 ± 3.61 ^A,ab^ | 17.57 ± 2.14 ^BC,b^ | 21.01 ± 2.97 ^A,ab^ | 22.68 ± 1.19 ^A,ab^ | 9.93 ± 0.04 ^A,c^ | 12.05 ± 0.22 ^AB,c^ |  |
|  | 2 | 25.71 ± 0.21 ^A,a^ | 23.03 ± 1.21 ^A,ab^ | 22.62 ± 2.76 ^A,ab^ | 21.52 ± 0.70 ^A,b^ | 24.27 ± 0.95 ^A,ab^ | 10.67 ± 1.85 ^A,c^ | 13.49 ± 2.47 ^AB,c^ |  |
|  | 3 | 23.82 ± 0.44 ^A,a^ | 22.01 ± 3.12 ^A,a^ | 22.20 ± 0.76 ^AB,a^ | 22.30 ± 0.56 ^A,a^ | 23.17 ± 0.18 ^A,a^ | 10.47 ± 3.32 ^A,b^ | 13.84 ± 0.81 ^AB,b^ |  |
|  | 4 | 25.92 ± 0.66 ^A,a^ | 21.63 ± 0.98 ^A,ab^ | 24.19 ± 1.40 ^A,a^ | 22.04 ± 0.62 ^A,ab^ | 24.78 ± 6.69 ^A,a^ | 8.13 ± 3.87 ^A,c^ | 15.43 ± 2.62 ^A,bc^ |  |

^1^ B sample prepared without inocula nor food additives (blank), C sample prepared with inocula, but without food additives (control), TA sample prepared with both inocula and 0.5% tartaric acid, PS sample prepared with both inocula and 0.1% potassium sorbate, SB sample prepared with both inocula and 0.06% sodium benzoate, GL sample prepared with both an inocula and 1.0% glycine, NA sample prepared with both inocula and 0.1% nicotinic acid.
^2^ Fermentation period.

^3^ ND: Not detected.

^4^ Values represent mean ± standard deviation determined by triplicate experiments. Mean values in the same column of the same BA on different days followed by a different capital letter (A-D) are significantly different (*p* < 0.05). Mean values in the same row of different samples on the same day followed by a different small letter (a-e) are significantly different (*p* < 0.05).
